# Chronic low back pain and physical activity among patients within the Brazilian National Health System: a cross-sectional study

**DOI:** 10.1590/1516-3180.2019.0312.R1.19112019

**Published:** 2020-06-01

**Authors:** Everton Alex Carvalho Zanuto, Rômulo Araújo Fernandes, Bruna Camilo Turi-Lynch, Robson Chacon Castoldi, Luana Carolina de Morais, Pedro Victor Tonicante da Silva, Jamile Sanches Codogno

**Affiliations:** I PhD. Physiotherapist/Physical Educator and Teacher, Department of Physical Education, Universidade do Oeste Paulista (UNOESTE), Presidente Prudente (SP), Brazil.; II PhD. Physical Educator and Teacher, Department of Physical Education and Postgraduate Physiotherapy Program, Faculdade de Ciências e Tecnologia (FCT), Universidade Estadual Paulista (UNESP), Presidente Prudente (SP), Brazil.; III PhD. Physical Educator, Department of Physical Education, Universidade Estadual Paulista (UNESP), Rio Claro (SP), Brazil.; IV PhD. Physical Educator and Teacher, Department of Physical Education, Universidade do Oeste Paulista (UNOESTE), Presidente Prudente (SP), Brazil.; V MSc. Physical Educator and Doctoral Student, Faculdade de Ciências e Tecnologia (FCT), Universidade Estadual Paulista (UNESP), Presidente Prudente (SP), Brazil. Author’s Title: MSc; VI Undergraduate Student, Department of Physical Education, Universidade do Oeste Paulista (UNOESTE), Presidente Prudente (SP), Brazil.; VII PhD. Physical Educator and Teacher, Department of Physical Education and Postgraduate Physiotherapy Program, Faculdade de Ciências e Tecnologia (FCT), Universidade Estadual Paulista (UNESP), Presidente Prudente (SP), Brazil.

**Keywords:** Adult, Public health, Low back pain, Exercise, Risk factors, Physical activity, Chronic low back pain, Alternative intervention

## Abstract

This study was based on filling a gap in our knowledge regarding the issue of what the protective effect of physical exercise on patients within the Brazilian National Health System could be, in relation to low back pain.

**OBJECTIVES:**

To determine the prevalence of chronic low back pain (CLBP) and to analyze the protective effect of physical exercise among patients over 50 years old attended at primary healthcare units (PHUs).

**DESIGN AND SETTING:**

Analytical cross-sectional study at Universidade Estadual Paulista (UNESP) that was conducted in two PHUs (Parque Cedral and Vila Real), located in different regions of the city of Presidente Prudente, Brazil.

**METHODS:**

In total, 327 patients were interviewed and evaluated at which retrospective characteristics covering the previous 12 months. The Nordic questionnaire was used to classify CLBP, and the Baecke questionnaire for physical activity level. The body mass index (kg/m^2^) was calculated using body mass and height values, both collected at the time of the interview.

**RESULTS:**

High prevalence of low back pain was found; 175 patients (53.5%) reported having had at least one episode of low back pain in the previous year. Of these, 71 (21.7%) answered yes to all four questions on the Nordic questionnaire and were classified as CLBP. Physical exercise remained associated with CLBP, independent of other factors (odds ratio = 0.35; 95% confidence interval = 0.15-0.80).

**CONCLUSION:**

High prevalence of low back pain was identified among PHU users. Physical exercise was associated as an independent protective factor against this pathological condition.

## INTRODUCTION

Low back pain has been found to be highly prevalent worldwide, for example in Germany (59%),^1^ Turkey (51%),^2^ France (55.4%)^3^ and the United States (50%).^4^ This health impairment can evolve to motor incapacitation, thereby severely compromising the quality of life of the individuals affected. Only 15% of the cases are of known etiology, which makes treatment difficult and reduces the chances of a good prognosis.^4^

Occurrences of low back pain are the second largest cause of medical consultations in the world,^4^ and this has a significant impact on public health systems, both through direct expenditure (consultations, medications, physiotherapy and examinations) and through indirect expenditure (reduction in productivity and absenteeism).^5^ In the United States, the estimated cost of chronic low back pain is 100 billion dollars per year,^6^ while in the United Kingdom the cost of back pain reaches 480 million pounds per year.^7^

A large part of the adult population suffers from this disorder (50.2%), of which 11.3% suffer from chronic low back pain.^8^ Consequently, low back pain leads the ranking in disability pensions (29.96 per 100,000 taxpayers).^9^ The following determinants of low back pain have been recognized: sex, chronological age, work condition, excess weight and sleep.^8^ Physical activity, although not clearly understood, seems to be a great ally in prevention of this pathological condition, especially because this is a non-pharmacological tool with low cost.^10^^,^^11^

Primary healthcare units (PHUs) serve the majority of the Brazilian population. They form the main triage locations for primary care, in which the focus is prevention and avoidance of the need for patients to access other levels of healthcare. Public policies need to be based on scientific evidence, so as to effectively meet the requirements of the community.

## OBJECTIVE

The aims of the present study were to determine the prevalence of chronic low back pain and to analyze the possible protective effect of physical exercise in different domains, among patients over 50 years of age who were attended at PHUs in Presidente Prudente, Brazil.

## METHODS

### Design and setting

This was an analytical cross-sectional study at Universidade Estadual Paulista (UNESP) that was conducted in two PHUs (Parque Cedral and Vila Real), located in different regions of the city of Presidente Prudente, São Paulo, Brazil.

### Ethical issues

This study was approved by the local Research Ethics Committee, at the Presidente Prudente campus of UNESP (procedural number: 241291; date: April 5, 2013).

### Sample calculation and subject selection process

The sample was composed of adults over 50 years old, of both sexes, who were attended at two PHUs (Parque Cedral and Vila Real), which are located in different regions of the city of Presidente Prudente. This age group was chosen because individuals of this age present greater incidence of chronic low back pain.^8^ The PHUs involved in the study were indicated by the Health Department of Presidente Prudente. This city is located in the west of the state of São Paulo and has approximately 208,000 inhabitants and a human development index of 0.806 (which is considered high).

The minimum sample size was calculated using the prevalence of chronic low back pain (14.7%) that was reported by Almeida et al.^12^ in an epidemiological study in the city of Salvador, Bahia, and a standard error of 5%. These were inserted into an equation for population parameters. In addition, a population of 208,000 inhabitants was considered, with a 95% confidence interval (95% CI) (z = 1.96). With the configuration described above, the equation indicated that interviews with at least 206 adults of both sexes would be needed. Lastly, 50% was added to allow for possible losses during the 12-month study period. This resulted in a need to interview 310 people, i.e. 155 patients at each BHU.

During the mornings of a 60-day period, all patients attended at these PHUs were invited to take part in the study, if they fulfilled all of the following inclusion criteria: i) registration at that PHU for at least one year, with at least one medical consultation attended over the previous six months; ii) age > 50 years; iii) resident in the city of Presidente Prudente for at least two years; iv) signing of an informed consent statement.

At each of the two PHUs selected, an initial screening of the medical schedules of all patients who had visited the unit over the previous thirty days was performed. At each PHU, all patients seen over the previous 30 days who met the inclusion criteria were invited to participate.

The patients thus selected were invited to attend the PHU for an evaluation and directed interview. The final sample consisted of 327 patients.

### Evaluations

#### Low back pain

The questionnaire developed by Kuorinka et al.^13^ and previously validated for the Portuguese language,^14^^,^^15^ which evaluates occurrences of musculoskeletal symptoms (pain, tingling or numbness), was used in this study but only for the lower back region. For each body region, there are four dichotomous questions (yes or no) regarding:

- the presence of musculoskeletal disorders over the previous 12 months;
- impairment of daily activities over the previous 12 months, due to these disorders;
- consulting a healthcare professional because of these disorders; and
- feeling the presence of these disorders in the week immediately prior to the interview. Positive responses to these four questions were considered to be indicative of chronic low back pain in the lumbar region.

### Economic condition

To determine the participants’ economic situation, a questionnaire developed by the Brazilian Association of Market Research Companies (2010) was used. In this, economic situation is subdivided from A (highest) to E (lowest). The sample was subsequently dichotomized into high economic situation (categories A and B) and low economic situation (categories C, D and E), as adopted by Fernandes et al.^16^ The present questionnaire was applied through an interview, which was conducted by the research coordinator.

### Nutritional status

Body mass index (in kg/m²) was calculated using body mass and height values, which were both obtained at the time of the interview, in accordance with the protocol of Lohman et al.^17^ The presence of overweight/obesity was diagnosed when the body mass index presented values between 25 and 29.9 kg/m² for overweight and ≥ 30 kg/m² for obesity.^18^

### Habitual physical activity

Information regarding habitual physical activity practices was collected by means of an interview using the questionnaire developed by Baecke et al.,^19^ which was translated and validated for Brazilian realities by Florindo et al.^20^ Through application of the instrument, it was possible to identify the level of habitual physical activity in each domain. The sum of the scores for each section gave the total score, i.e. habitual physical activity. For the purpose of statistical analysis, the sample was subdivided into quartiles according to the total physical activity score from the instrument (which analyzes three domains of physical activity: i- occupational; ii- physical exercise during leisure; iii- leisure and locomotion). Participants in the 75^th^ percentile and above (highest quartile) were considered physically active. Cycling was also computed dichotomously (yes or no).

### Confounding factors

The following were considered to be confounding factors: education ([i] 1-4 years; [ii] 5-8 years; [iii] 9-11 years; or [iv] ≥ 12 years); gender (male or female); age (< 65 years or ≥ 65 years); ethnicity (white, black or other color); and current occupational activity (yes or no).

### Statistical procedures

Numerical variables were presented as means and standard deviations, and the means were compared using Student’s t test. Categorical variables were expressed as absolute and percentage values, and univariate statistical tests were applied to these (χ^2^, with the Yates correction applied via 2 x 2 contingency tables). When significant differences were found, the values were inserted in a multivariate model (binary logistic regression) with hierarchical insertion. The magnitude of associations was expressed in terms of odds ratio (OR) values and their respective 95% confidence interval (CI). In our analysis model, associations between the dependent variable (chronic low back pain) and the independent variables were investigated, and the results were corrected for possible confounding variables (sex, economic situation, adiposity and age). The statistical analyses were performed using specific software (BioEstat version 5.0) and, in all procedures, the significance level adopted was 5%.

## RESULTS

The sample was composed of 327 patients, i.e. 229 females (70%) and 98 males (30%). The mean body mass index was 29.1 ± 5.4 kg/m², the mean age was 62 ± 8.8 years and 83 patients (25.4%) had a current occupation. Low back pain was found to be present in 175 patients (53.5%), i.e. at least one episode of low back pain in the previous year. Among these patients with low back pain, 71 (21.7%) gave positive responses to the four questions about low back pain and were therefore classified as having chronic low back pain. Females had higher odds of developing chronic low back pain (OR = 2.63; 95% CI = 1.24-1.55). The independent variables are presented in Table 1, thus characterizing the sample and the influence of these variables on the outcome of chronic low back pain.

**Table 1.** General characteristics of the sample

	Chronic low back pain*		P-value
	Yes	No	
Numerical variables (µ ± SD)			t-test
Age (years)	57.9 ± 6.9	63.1 ± 8.9	0.003
Weight (kg)	75.4 ± 15.3	71.5 ± 14.9	0.055
Height (m)	1.57 ± 8	1.57 ± 8.9	0.730
Body mass index (kg/m^2^)	30.5 ± 6.1	28.7 ± 5.2	0.025
Categorical variables n (%)			χ² test
Sex			
Female	60 (26.2)	169 (73.8)	0.004
Male	11 (11.3)	87 (88.7)	
Ethnicity			0.204
White	44 (20.5)	170 (79.5)	
Black	13 (18.8)	56 (81.2)	
Others	14 (31.8)	30 (68.2)	
Age			0.001
< 65 years	61 (28.8)	151 (72.2)	
≥ 65 years	10 (8.8)	105 (91.2)	
Body mass index			0.220
Normal	12 (16.0)	63 (84.0)	
Overweight/obese	59 (23.4)	193 (76.6)	

*Positive responses to the four questions in the questionnaire; µ ± SD = mean and standard deviation.

In the whole sample, 78 patients (23.9%) were considered physically active. The variables associated with chronic low back pain were inserted into the binary logistic regression, which resulted in the odds ratios presented in Table 2.

**Table 2.** Factors associated with chronic low back pain among Brazilian primary healthcare unit patients

	Chronic low back pain*		χ²	Crude OR (95% CI)
	Yes n (%)	No n (%)	P-value	
Cycling			0.02	
Yes	4 (8.3)	44 (91.7)		0.291 (0.101-0.839)
No	67 (23.8)	212 (76.2)		1.00
Exercise			0.04	
Yes	10 (12.8)	68 (87.2)		0.453 (0.220-0.935)
No	61 (24.5)	188 (75.5)		1.00
Work			0.02	
Yes	26 (31.3)	57 (68.7)		2.010 (1.146-3.551)
No	45 (18.4)	199 (81.6)		1.00

*Positive responses to the four questions in the questionnaire; OR = odds ratio; 95% CI = 95% confidence interval.

The variables that were associated with chronic low back pain, as shown in Table 2, were inserted hierarchically in multivariate models, as demonstrated in Table 3, to test whether these associations were independent of the other factors.

**Table 3.** Model adjusted for association between chronic low back pain and independent variables

	Model 1	Model 2*	Model 3**
	OR (95% CI)	OR (95% CI)	OR (95% CI)
Cycling			
Yes	0.284 (0.094-0.861)	0.283 (0.093-0.859)	0.378 (0.122-1.167)
No	1.00 (Reference)	1.00 (Reference)	1.00 (Reference)
Physical exercise			
Yes	0.390 (0.182-0.836)	0.388 (0.181-0.831)	0.356 (0.159-0.800)
No	1.00 (Reference)	1.00 (Reference)	1.00 (Reference)
Work			
Yes	1.308 (0.715-2.393)	1.302 (0.711-2.382)	1.181 (0.628-2.220)
No	1.00 (Reference)	1.00 (Reference)	1.00 (Reference)

OR = odds ratio; 95% CI = 95% confidence interval; yes ≥ 75^th^ percentile; no < 75^th^ percentile; Model 1: model adjusted according to socioeconomic variables (sex, age, ethnicity and economic situation); Model 2: model adjusted according to socioeconomic variables and body mass index; *Hosmer and Lemeshow test with P-value = 0.544; Model 3: all variables inserted; **Hosmer and Lemeshow test with P-value = 0.061.

## DISCUSSION

The prevalence of low back pain found in the present study (53.5%) is similar to that reported from another survey carried out in the same city (50.2%),^8^ and also to reports from other studies around the world.^1^^,^^2^^,^^3^^,^^4^ However, the prevalence of chronic low back pain, of 21.7%, was higher than what had been found in other studies conducted in Brazil: in the states of Bahia (14.7%)^12^ and São Paulo (11.3%)^8^. It is possible this difference is due to the fact that these other studies were population-based studies, thus differing from the characteristics of the population of the present study, which comprised PHU patients. Another important factor that contributed towards higher prevalence of low back pain was the age group evaluated in the present study, i.e. individuals over 50 years old. This differs from population studies, which encompass all adults over the age of 18 years. This important observation reflects the need for prophylactic measures and/or specific treatments to control this pathological condition. This need is not exclusive to the present study.

In the models created, female sex was shown to be a risk factor for the development of chronic low back pain, independently of the other confounding factors (OR = 2.63; 95% CI = 1.24-1.55). This greater occurrence may have been due to anatomical-functional differences, but may also have come from women’s double working day.^8^ Sex lost statistical significance only when physical and occupational activities were inserted in the model. Culturally, in Brazil, men are more active during leisure time than women,^16^ and this would be a protective factor against chronic low back pain. On the other hand, men perform functions with greater physical overloads, which increases the risk of developing chronic low back pain.^3^ This factor may be the explanation for the loss of significance when the data were adjusted for these variables.

Patients who reported that they were working also presented greater risk of developing chronic low back pain (OR = 2.01; 95% CI = 1.14-3.55). This association lost significance when inserted into the multivariate model together with age and economic situation, perhaps because people in a higher economic situation have better information about healthy life habits and also do not need to work when they have reached this age group.^1^

It is known that workers in several types of occupation perform functions that contribute towards installation of this pathological condition. Moreover, chronic low back pain can cause decreased productivity and quality of life among the individuals affected, and this may lead to susceptibility to absences from work. It is therefore important to stress that management teams in public institutions and other employers need to make efforts to prevent and/or combat this important health problem.^1^^,^^22^^,^^23^

Encouragement to practice physical activity may be a strategy, through advertisements that incorporate healthy values and increase the level of physical activity. In Brazil, the example of the United States could be adopted, with a national road map to improve the quality of health through a physically active lifestyle, like the one developed by the American College of Sports Medicine, which involves four steps: awareness, education, partnerships and monitoring.^24^

The physical activity practices among PHU users seem to be a relevant problem, since only 24% of the interviewees were classified as sufficiently active. This is a low percentage and is similar to that reported by Turi et al.^25^ in the city of Bauru, also among PHU patients (24.9%). In Brazil, promotion of physical activity only began widely in the 1970s.

Promotion of physical activity has greater efficacy among younger individuals. Moreover, if physical activity practices are insufficient during youth, this tends to continue into adulthood, since the characteristics of these practices tend to stabilize over time.^26^^,^^27^ Given that the population of this study was older than 50 years, the participants had probably not been given as much encouragement as young people currently are, which will have led to higher rates of sedentary behavior among people who are now over 50 years of age.^28^

Lastly, the greatest contribution of the present study was to make a correlation between the level of physical activity and occurrences of chronic low back pain. Physically active people were found to have 65% less chance of developing chronic low back pain, independently of other factors. This differed from some previous studies, in which physical activity lost its association when inserted in the multivariate model.^8^^,^^12^ However, the current findings corroborate those of other studies, in which the benefits of being physically active on this disorder were demonstrated.^1^^,^^22^^,^^23^ This validates the practice of physical activity and makes it an important option in relation to prevention and/or treatment of this disorder.

The beneficial effects of regular physical activity on maintenance of good health have been recognized worldwide.^24^^,^^28^ In relation to chronic low back pain, these effects are no different: physical activity plays a fundamental role, indirectly or directly, in the preventive aspects of reducing the risks of developing this pathological condition.

There is evidence indicating that physically active people are less likely to develop poor sleep quality. Poorer sleep quality has been correlated with chronic low back pain.^8^ Another indirect benefit of physical activity to reduce the risks of developing chronic low back pain is in controlling obesity.^8^^,^^11^^,^^13^ Systematized general physical exercise programs of both low and moderate intensities are considered to be directly protective against chronic low back pain, and are applied in treating this disorder.^11^

It is worth highlighting the methodological design of the present study. It used a sample of the population that was of significant size and demonstrated that physical activity was a protection factor in different domains of leisure and locomotion, in relation to chronic low back pain. Thus, public policies aimed towards increasing the level of physical activity should be encouraged, such as inclusion of physical educators and training infrastructure within PHUs.

However, there were some limitations to the present study. The lack of a more sophisticated method for diagnosing low back pain, the reverse causality bias applied in this study and the data collection method used (active charts alone), which led to a large discrepancy in the numbers of men and women in the sample, should be considered to be the main limitations. These form directions for future studies.

## CONCLUSION

A high prevalence of low back pain was identified among PHU patients, while the practice of physical exercise during leisure time was a protective factor against this outcome.
